# A high-throughput RNA-seq approach to profile transcriptional responses

**DOI:** 10.1038/srep14976

**Published:** 2015-10-29

**Authors:** G. A. Moyerbrailean, G. O. Davis, C. T. Harvey, D. Watza, X. Wen, R. Pique-Regi, F. Luca

**Affiliations:** 1 Wayne State University, Center for Molecular Medicine and Genetics, Detroit, 48201, USA; 2 University of Michigan, Department of Biostatistics, Ann Arbor, postcode, USA; 3 Wayne State University, Department of Obstetrics and Gynecology, Detroit, 48201, USA

## Abstract

In recent years RNA-seq protocols have been developed to investigate a variety of biological problems by measuring the abundance of different RNAs. Many study designs involve performing expensive preliminary studies to screen or optimize experimental conditions. Testing a large number of conditions in parallel may be more cost effective. For example, analyzing tissue/environment-specific gene expression generally implies screening a large number of cellular conditions and samples, without prior knowledge of which conditions are most informative (e.g., some cell types may not respond to certain treatments). To circumvent these challenges, we have established a new two-step high-throughput RNA-seq approach: the first step consists of gene expression screening of a large number of conditions, while the second step focuses on deep sequencing of the most relevant conditions (e.g., largest number of differentially expressed genes). This study design allows for a fast and economical screen in step one, with a more efficient allocation of resources for the deep sequencing of the most biologically relevant libraries in step two. We have applied this approach to study the response to 23 treatments in three lymphoblastoid cell lines demonstrating that it should also be useful for other high-throughput transcriptome profiling applications requiring iterative refinement or screening.

In the field of transcriptomics, a variety of study designs could take advantage of a strategy that tests a large number of conditions prior to further analysis of relevant ones. Examples of such applications include time-course experiments (e.g., Amit *et al.*^1^), with an initial screen over a large number of timepoints; population-specific response profiling (e.g., Maranville *et al.*^2^), with a large number of treatments performed in a few individuals; and large scale sh-RNA studies (e.g., Cusanovich *et al.*^3^). In each of these cases, deep sequencing can then be used to characterize the transcriptome only in the most relevant conditions (i.e. timepoint, treatment, tissue type). When interested in understanding the regulatory mechanisms underlying cellular response to environmental perturbations, current experimental setups are costly and laborious and focus only on the analysis of one particular cellular environment. One of the few examples of studies considering more than one cellular environment is the Connectivity Map initiative^4^, which characterized the transcriptional response to 164 small-molecule perturbagens in four cancer cell lines using microarray technology. Similar projects could now instead use RNA-seq technology for the initial screening step with many practical advantages when transitioning to a focused analysis of the most relevant conditions (e.g., isoform quantification, identifications of new transcripts, allele-specific expression, and changes in lowly expressed genes or long non-coding RNAs).

Since the development of RNA-seq^5^,^6^,^7^, a variety of protocols have been introduced to measure transcript expression and investigate specific biological problems. For example, direct RNA sequencing^8^ allows sequencing of RNA molecules skipping cDNA synthesis and can thus analyze short, degraded and/or small quantity RNA samples. Another example of fast and automatized RNA-seq protocols is the Tn-RNA-seq^9^ approach, which uses transposase-based incorporation of sequencing adapters in cDNA libraries. Most RNA-sequencing studies that only require gene expression quantification are currently collecting tens of millions of reads per sample. A recent report by Hou *et al.*^10^, however, shows that gene expression for highly abundant transcripts can be reliably quantified with less than five million reads per library. Similarly, recent theoretical predictions and empirical findings demonstrated that shallow mRNA sequencing at extremely low depths may be useful for other applications (e.g., Kliebenstein *et al.*^11^). Pollen and colleagues^12^, for example, have shown that tissue identity from single cells transcriptome analysis can be detected with a shallow sequencing depth of 10,000 reads/single cell, while specific gene expression signatures require at least 50,000 reads/cell. Here we investigated whether a shallow sequencing approach can be used as an initial screening step of differential gene expression, to be followed up with deep sequencing of the most informative libraries.

To this end we have developed a cost-effective two-step strategy that uses the ability to index and pool many (96 or more) RNA-seq libraries in parallel, and it can be used in combination with any RNA-seq technique as long as it allows for multiplexing. This strategy allows the researcher to rapidly screen a large number of sample conditions and strategically allocate sequencing resources for in depth analysis only of the relevant cases. We demonstrate this approach by exploring the transcriptional response to a wide panel of environmental perturbations (23 treatments) in three lymphoblastoid cell lines (LCLs) samples. The results show that our approach should also be applicable to similar scenarios requiring high throughput screening across multiple cell lines, treatments, time points and/or patient samples in a variety of contexts, such as: population genetic studies, parallel shRNA knockdowns, mutagenesis screens, pharmacological drug testing, stem cell differentiation monitoring and cancer transcriptome profiling.

## Results

### The two-step approach

**Figure 1:**
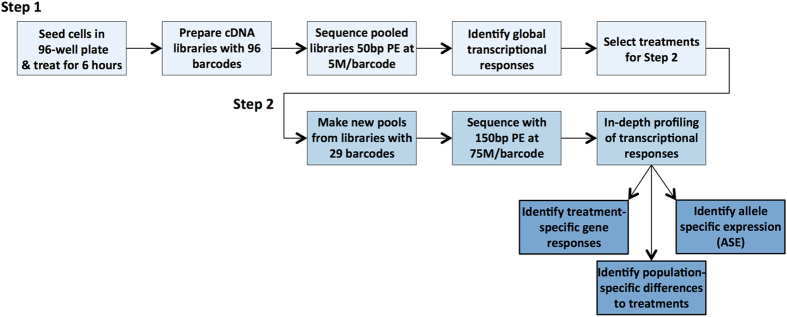
Workflow of the two-step approach.

Figure 1 presents an outline of the new high-throughput two-step RNA-seq approach we have developed. In step one we characterize global changes in gene expression. Here we used a modified RNA-seq protocol (see Methods) better suited for our specific application, but similar results can be achieved with popular commercial RNA-seq kits that allow for high multiplexing (96-well plate format) such as the Illumina TruSeq Stranded mRNA HT Sample preparation kit or the NEBNext Ultradirectional (NEB) library preparation kit. Many of these commercially available kits can work with liquid handling robots that automatize the majority of the experimental steps (e.g., Beckman Coulter Biomek FXp, Eppendorf epMotion 5075, and others).

In the first step, all samples are experimentally processed in parallel, from tissue culture and treatments to library preparation, thus minimizing experimental variation from testing hundreds of conditions at the same time. Additionally, high multiplexing allows reducing the number of controls that would need to be repeated across different treatment batches in a less multiplexed experimental setup (e.g., 93 treatments plus 3 controls). A 96-libraries pooling and shallow sequencing strategy is then used to minimize the amount of resources used in the screening step. Here we demonstrate that shallow sequencing depth (<10 M reads) allows detecting global and biologically relevant gene expression changes and can be used to identify relevant conditions to follow up in step two. Even for study designs that require deep sequencing of large number of samples (e.g., 96), our two-step approach allows using the first step to QC the libraries, before investing in deep sequencing efforts.

For the second step, we do not need to prepare new libraries, and we can simply repool a selection of the initial libraries, without additional experimental costs. Additionally, we can optimize library concentrations to pool in order to achieve even representation of individual libraries. This is done by calculating a digital library concentration from the sequencing run performed in step one. Note that this digital library concentration is the fraction of reads from the total sequenced in the pool, and it naturally takes into account potential differences across the libraries in sequencing output (e.g., due to flow cell cluster formation in Illumina sequencing machines) (see Methods, Equation 1). Even in situations where deep sequencing data are to be collected for all samples, using a two-step approach makes possible to repool the samples to achieve a more uniform allocation of sequencing reads across samples. As a result, in many applications, using the same budget, greater sequencing depth can be allocated to step two instead of step one. Below we present an application of the two-step approach to analyze the response to 23 environmental perturbations in LCLs.

### Step one: Identifying global changes in gene expression from low-coverage data

**Figure 2:**
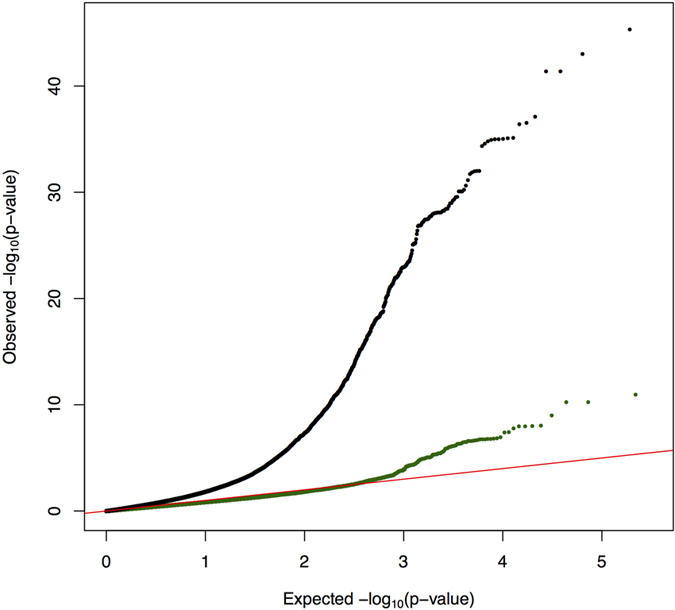
QQplot of the *p*-value distribution for DE genes in response to dexamethasone (black) and estrogen (green), compared to the expectation under a uniform distribution (red). Additional QQ plots from step one and step two are available in the supplements.

To characterize the response to treatments, cells were treated with the panel of treatments listed in Table 1 for 6 hours. Cells from all treatment conditions, including the vehicle controls, were cultured and harvested in parallel at the same time point, thus allowing for a better control of technical noise, or biological variation that is independent of the treatment. For example, this design controls for temporal changes in gene regulation that are independent of the treatment (e.g., changes in cell cycle phase over time, reagent batch effect) but otherwise could be confounded or add noise to the measurements. To achieve greater confidence and accuracy to measure baseline gene expression, for each LCL sample, the control treatments were performed in triplicates. For all stages of sample preparation we have used a 96-well plate study design (3 samples and 32 treatment conditions, Figure S1) from cell culturing to RNA extraction and library preparation, thus facilitating increased sample processing throughput. To identify differentially expressed (DE) genes we used the method implemented in the software DEseq2^13^, which estimates variance-mean dependence in the read counts for each gene and tests for differential expression based on a model using the negative binomial distribution. Each treatment was matched to the appropriate vehicle control (Table 1) for this analysis. However, when comparing pairs of controls to each other we did not detect any DE genes (10% Benjamini-Hochberg controlled FDR^14^ [BH-FDR], Figure S2). To assess the calibration of the tests for differential expression on low coverage data, we used QQ-plots and compared the *p*-value distribution from DESeq2 to the expected uniform distribution. We observed that in most cases the tests are well calibrated (Fig. 2 and Figure S3).

**Figure 3:**
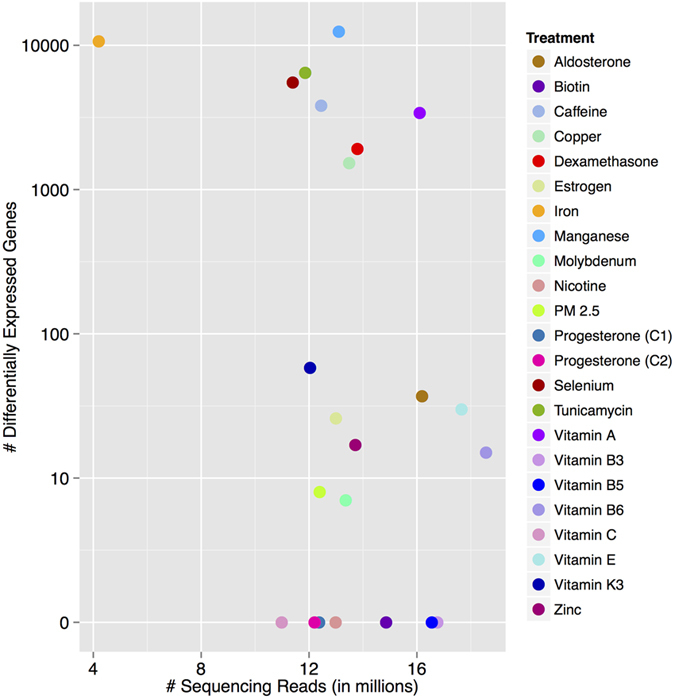
DE genes and sequencing depth. Scatterplot of DE genes (10% BH-FDR) versus the sequencing depth for each shallow sequencing treatment. The reported sequencing depth is the total number of reads across the three individuals.

We next asked whether our ability to identify the conditions with strongest differential expression may depend on sequencing depth. Figure 3 shows that in the context of the expected variations in sequencing depth from multiplexing of samples, the strongest conditions stand out even if they were sequenced at relatively lower depths than other conditions. For example, we identified thousands of DE genes for iron and tunicamycin, which are among the treatments with less coverage. On the other hand, we detected <100 DE genes in response to vitamin B6 and vitamin E, even though these are the treatments for which we collected the largest number of reads. Prior experiments performed by our group (see Luca *et al.*, 2013^15^, Maranville *et al.*, 2011^16^) showed that dexamethasone induces a strong transcriptional response in LCLs, while estrogen doesn’t have a strong impact on gene regulation in this cell type. After running DESeq2, we identified 1,919 DE genes in response to dexamethasone, while only 26 DE genes were detected in cells treated with estrogen, thus confirming previous results (Fig. 2).

**Figure 4:**
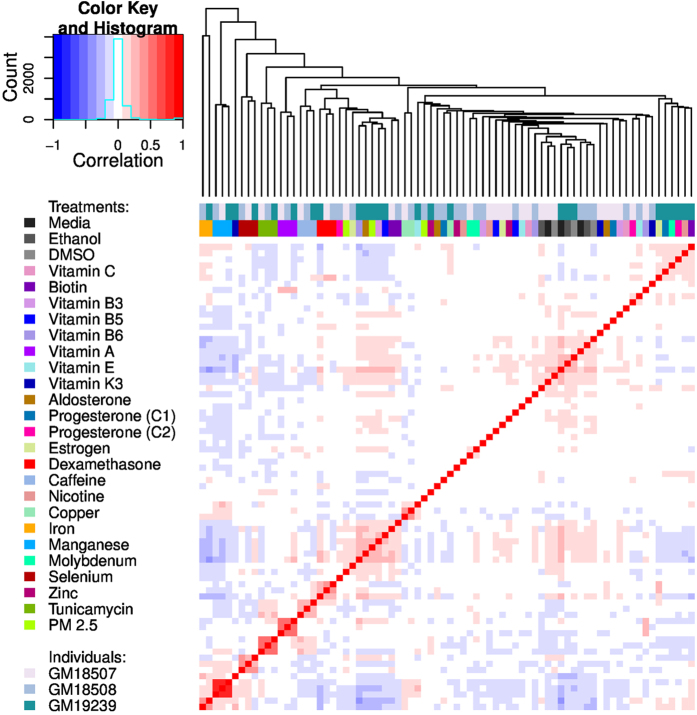
Heatmap and hierarchical clustering of gene expression levels across all shallow sequenced samples. Gene expression levels (FPKMs) were obtained for each sample (individual X treatment combination) as a vector indexed by gene. Those vectors were clustered using hierarchical clustering and the dendrogram is displayed at the top of a heatmap visualizing the Pearson correlation between each pair of samples. The sample identity is detailed by a two-way coloring indexing the individual and treatment (see legend).

To identify major similarities and differences in the transcriptional response to our panel of treatments, we performed hierarchical clustering on the transcript expression data for each treatment (expressed in FPKMs). Figure 4 shows a heatmap of the correlation matrix across all treatment conditions and samples. Some key features appear evident even with low sequencing depth: control samples cluster together; treatments that induce a strong response are distinct from all other treatments and controls, and show a clear pattern where the three samples for each treatment condition cluster very tightly.

Given the high number of DE genes observed for certain treatments, we asked whether they could indicate that the cell is undergoing a cytotoxic response. To this end we compared the transcriptional response from RNA-seq data to the cytotoxic response measured in viability assays. To measure cell viability we used the Promega Glo-Max assay, and compared ATP production measured in relative luminescence units in treatment and control cells. We observed a significant negative correlation between number of DE genes and cell viability after 48 hrs treatment (Spearman *ρ* = −0.52, *p* = 0.01, Figure S4). This suggests that when an extremely large number of DE genes is observed, it is indicative of major changes in the cell physiological state, which ultimately may lead to cell death. For example, the largest number of DE genes was observed for treatments such as iron (10,639) and manganese (12,445), which were administered at supra-physiological doses (Figure S3).

Overall the results from step one show that even from low sequencing depth data it is possible to identify biologically meaningful global changes in gene expression that are relevant to assess the cellular response to environmental perturbations.

### Step two: Following up the most relevant conditions

The information collected in step one of our approach can be most effectively used to re-pool individual libraries by selecting the treatment conditions biologically relevant for the system under-study. As a proof of principle, we selected four treatment conditions, vitamin A, copper, iron, and selenium, for deeper sequencing (75 M reads/sample) in all three cell lines to investigate the transcriptional response to these environmental perturbations with greater resolution.

**Figure 5:**
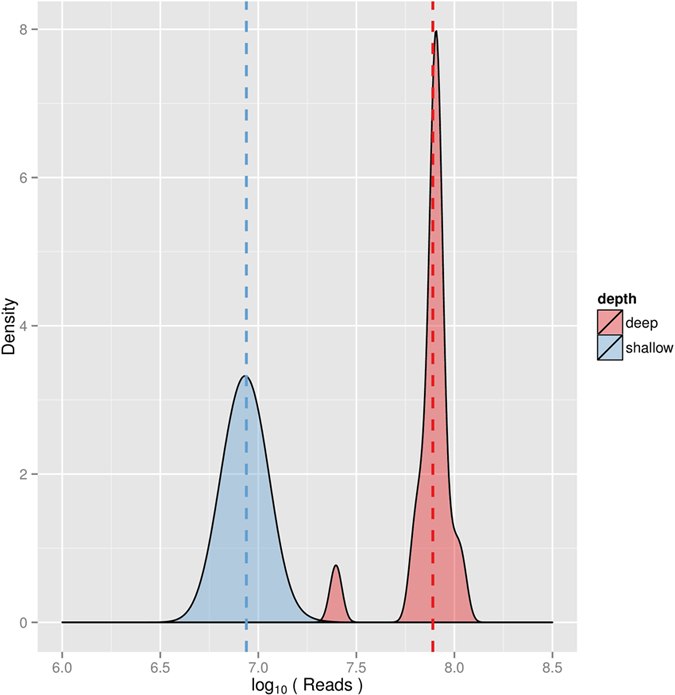
Density plot of raw (unfiltered) sequencing depth across individual barcoded samples for the shallow sequencing (blue) and deep sequencing (red) runs. Note that in step two, we aimed to collect approx 75 M reads for each treatment sample, while for the control samples (e.g., EtOH) we aimed to collect 75 M reads across the three technical replicates (approx 25 M reads per replicate), in order to achieve even representation of sequencing depth across treatment conditions. In this plot reads for each control sample were pooled across technical replicates. Only for the iron samples we did not aim for 75M reads as we noticed the cytotoxic effect and decided not to pursue much further. Dotted lines indicate the average sequencing depth in each step.

One of the challenges when sequencing highly multiplexed pools of libraries is achieving similar depth of coverage across samples. Figure 5 shows density plots of sequencing depth across shallow and deep sequencing samples. The distribution of sequencing depth is a function of factors related to the sequencing technique and instrument (for example, efficiency in cluster generation on an Illumina sequencer). For this reason, it is possible to account for these factors when determining pooling concentrations for the deep sequencing pool. We developed a formula (Equation 1) that uses information from the low coverage data to learn about “read” concentration per library and also accounts for the sequencing output of each individual sequencing run (see Methods). This is much better than any standard library quantification approach, because we have a “digital” count of the actual reads that contribute to generate clusters on the flow-cell per unit of volume of the library. As expected, in step two, we observe a much tighter distribution of sequencing depths across samples.

**Figure 6:**
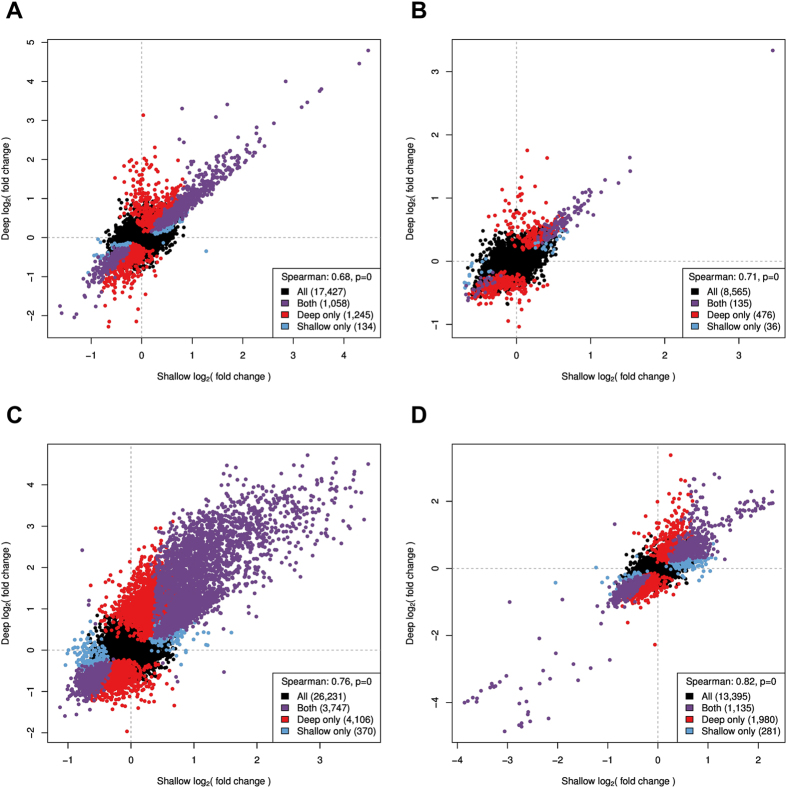
Correlation in the transcriptional response between shallow and deep sequencing. Plotted is the log_2_(fold change) for each gene calculated from shallow and deep sequencing data for the four treatments analyzed in step two. Colored points represent genes differentially expressed at 1% BH-FDR. Vitamin A (**A**), copper (**B**), iron (**C**), selenium (**D**). Spearman’s *ρ* (legend) is calculated using all genes.

**Figure 7:**
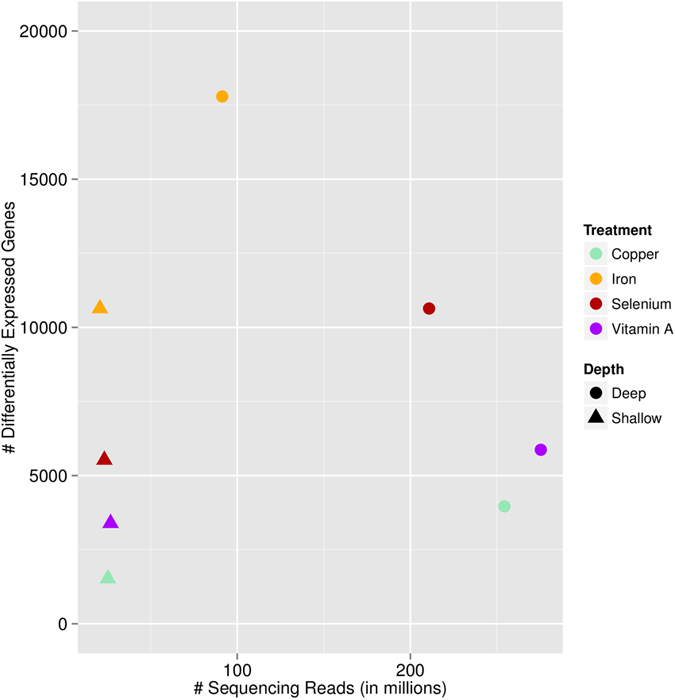
Comparison of sequencing depth and number of differentially expressed genes (10% BH-FDR) between shallow and deep sequencing runs. The reported sequencing depth is the total number of reads across the three individuals.

We then used DEseq2 to identify DE genes in the deep sequenced libraries. Table 2 shows the number of DE genes, and their direction of expression change. We found that expression fold change is highly correlated between the shallow and deep sequencing experiments for the same treatment (Spearman *ρ* > 0.7, Fig. 6, Figure S5, Figure S6), which confirms that gene expression changes detected from shallow sequencing can be used to identify biologically relevant treatments for follow up studies. The small subset of DE genes identified only in the shallow sequencing data are most likely false positives due to larger uncertainties in the fold change estimates. Accordingly, we find larger standard errors (0.006 - 0.02 higher) for the log fold changes in the shallow data, compared to the deep sequencing data, for all treatment conditions except iron (0.003 lower) (Figure S7). We found minimal changes in transcript length (100bp - 350bp difference) and average GC content (0.04% - 0.6% difference) between DE genes identified from shallow or deep sequencing. No major differences are detected in gene expression levels derived from shallow and deep sequencing (Figure S6) due to a run effect as samples cluster by individual rather than sequencing run. As expected, with deep sequencing data we can identify transcriptional changes with greater sensitivity at the same BH-FDR level. Figure 7 shows the increase in number of DE genes as a function of sequencing depth from step one to step two.

**Figure 8:**
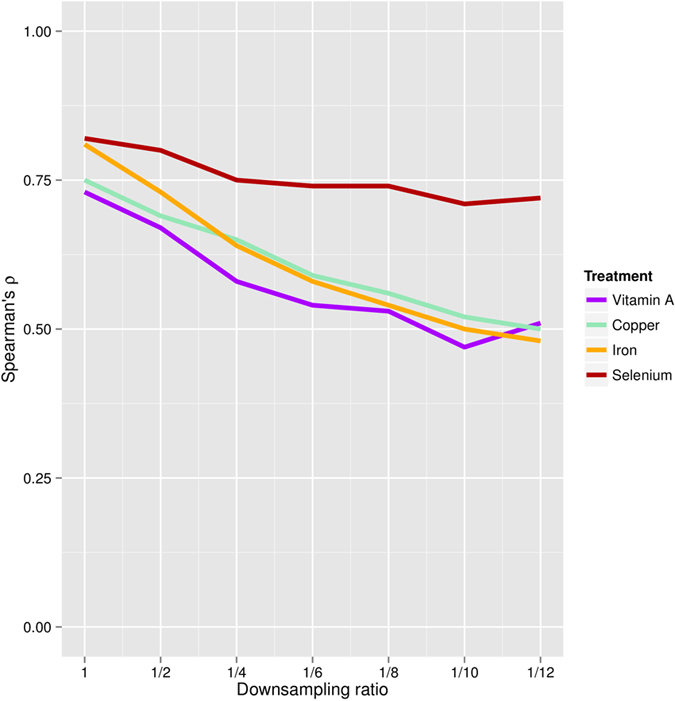
Correlation of downsampled shallow sequencing runs (step one) to deep sequencing data (step two). The DEseq2 log fold-change obtained after step two are correlated (Spearman correlation) with the fold changes obtained at step one for different downsampling ratios (1, 1/2, 1/4, 1/6, 1/8, 1/10 and 1/12), that would correspond to multiplexing higher number of samples (96, 192, 384, 576, 768, 960 and 1152).

To investigate to which extent we can decrease the amount of sequencing performed in step one, we downsampled reads from shallow sequencing runs to simulate the effects of using a lower coverage for the first step (from the proposed 96 up to 1152 multiplexed samples). Figure 8 and Figure S8 show the correlation between fold changes at downsampled depths with fold changes from step two. Although the number of significantly DE genes may decay more rapidly as we multiplex more samples, the correlation with deep sequencing is >0.5 when we subsample down to 1/8, corresponding to multiplexing 768 samples. The observed correlations support the possibility of using higher multiplexed study designs.

### In depth analysis of gene regulation in response to environmental perturbations from step two

To investigate similarities in the transcriptional response to the four treatments that were deep sequenced, we calculated pairwise Spearman rank correlations on the transcript fold change. We observed that responses to metal ions (copper, iron, selenium) tend to be more highly correlated with each other than to vitamin A. The highest correlation was observed between copper and iron (0.43, *p* < 10^-16^). This suggests that LCLs respond to these treatments through similar gene regulatory pathways.

To further investigate the regulatory pathways altered during the response to these treatments, we performed GO (Gene Ontology) enrichment analysis using the DAVID online tool^17^ and focusing on biological processes (5% BH-FDR, Supplementary Tables S1–S8). We observed that upregulated genes in response to vitamin A are enriched for the immune response and related processes (e.g., leukocytes and lymphocytes activation), which is in line with the known role of vitamin A as an activator of immune function^18^. Upregulated genes in response to copper are enriched for genes involved in the protein ubiquitination biological processes, and the same result is observed for upregulated genes in response to selenium. This supports the observation that these two metal ions elicit very similar transcriptional responses, which are clearly distinct from the response induced by treatment with vitamin A. However GO enrichment analysis also points to an anti-inflammatory role for selenium, as down-regulated genes in response to this metal ion are enriched for leukocytes activation. Finally, genes upregulated in response to iron are enriched for metal ion transport and cell-cell adhesion among the top biological processes, while down-regulated genes are enriched for RNA and DNA metabolic processes as well as key cellular processes such as mitosis. These last enrichments reflect the observed cytotoxicity of the iron treatment we performed on the cells.

## Discussion

We have developed a novel high-throughput and cost-effective approach to screen and analyze the transcriptional response to a large number of environmental perturbations through RNA-seq. This approach consists of two steps, where only the first step requires cell culture experiments and library preparation and allows for a fast and economical screen of a large number of environmental conditions that are followed up in the second step through deep sequencing of re-pooled libraries.

We have shown that shallow sequencing of 96 pooled libraries allows identifying, with minimal costs (approximately $60/sample, including library preparation and sequencing), the most interesting conditions while capturing biologically relevant and informative gene expression changes. This removes the burden of deep sequencing uninformative libraries in pilot studies. We have presented an application of this approach to analyzing 23 environmental perturbations and appropriate controls in three LCL samples. However, this approach can be successfully applied to other study designs where it is most economical to test a large number of conditions prior to further analysis of relevant ones. Examples of such applications include analysis of environmental perturbations, genotypic differences, disease states, and time course experiments.

The second step of our approach can be designed to achieve varying levels of sequencing depth and read length, depending on the question being asked. Here we have used step two to validate the shallow sequencing step and learn about transcriptional changes in response to three metal ions and a vitamin/nuclear receptor ligand treatments.

Given the significant savings allowed by step one, it is possible to invest in deep sequencing of step two pools to the degree necessary to answer specific biological questions. For example, using more cycles to get longer reads may facilitate transcript isoforms detection and quantification^19^. A sequencing depth of 80 M reads or above combined with longer reads also helps in identifying allele specific expression (ASE) even in the absence of genotype information, as our group has recently shown^20^.

With the availability of desktop sequencer instruments (such as the Illumina NextSeq500), this two-step protocol will allow for fast screening and in-depth analysis of relevant conditions in less than 1 week-time. Compared to microarray-based pilot studies with 96 samples, our approach allows for 40% savings (e.g., $9,600 with the least expensive microarray option vs $5,800), with subsequent optimal allocation of resources to meaningful biological conditions (in step two), thus reducing the amount of time and funds spent on unsuccessful pilot/exploratory studies. In our example application, for the same treatments we would consistently detect more than 50% of the originally differentially expressed genes (on average) even if we had multiplexed 2 times more samples (192 total) in step one. Thus this approach could be even more effective when a project requires higher number of samples.

## Methods

### Cell culture and treatments

Lymphoblastoid cell lines (LCLs) were purchased from Coriell Cell Repositories. Prior to the experiment, cells were cultured, at 37° and 5% CO_2_, in RPMI 1640 (Gibco), supplemented with 15% heat-inactivated fetal bovine serum and 0.1% Gentamycin. The following LCLs were used: GM19239, GM18507, and GM18508. LCLs were cultured in “starvation medium” composed of RPMI 1640, supplemented with 15% charcoal-stripped fetal bovine serum (CS-FBS) and 0.1% Gentamycin for four days. Cells were then treated, while in mid-log phase exponential growth, with the treatment panel (Sigma Aldrich) in Table 1 for 6 hours.

### Sample Collection and mRNA isolation

Treated cells were collected by centrifugation at 2000 rpm and washed 2× using ice cold PBS. Collected pellets were lysed on the plate, using Lysis/Binding Buffer (Ambion), and frozen at −80°. Poly-adenylated mRNAs were subsequently isolated from thawed lysates using the Dynabeads mRNA Direct Kit (Ambion) and following the manufacturer instructions.

### A modified RNA-seq library preparation protocol

We modified the NEBNext Ultradirectional (NEB) library preparation protocol to use 96 Barcodes from BIOOScientific added by ligation, this allowed us to reduce the overall library preparation cost to $47/sample. Specifically, RNA-seq libraries were prepared using the NEBNext ultradirectional library preparation protocol, with the following changes. RNA was fragmented at 94° for 5 minutes to obtain fragments 200–1500 bp in size. SPRI Select beads (Beckman Coulter) were used in all purification steps and size selection was performed to obtain 300–450 bp fragments. After the cDNA synthesis, to the 65 *μ*L of dA-Tailed cDNA were added the following components: 15 *μ*L of Blunt/TA Ligase Master Mix, 2.39 *μ*L of BIOO Scientific Barcode Adaptors (1–96), 1.11 *μ*L of Nuclease-free water. The samples were incubated for 15 minutes at 20° in a thermal cycler. USER Excision and PCR Library Enrichment were performed according to the following protocol. To the size selected cDNA (20 *μ*L) were added the following components: 3 *μ*L of NEBNext USER Enzyme, 25 *μ*L of NEBNext High-Fidelity PCR Master Mix, 2×, 2 *μ*L of BIOO Scientific Universal Primer (12.5 *μ*M). The individual libraries were quantified using the KAPA real-time PCR system, following the manufacturer instructions and using a custom-made series of standards obtained from serial dilutions of the phi-X DNA (Illumina). Pools of 96 samples from the first step were sequenced on two lanes of an Illumina HiSeq2500 in fast mode to obtain 50 bp PE reads, at the University of Chicago Genomics core. Alternatively, this could be run on one lane of the Illumina Next-Seq 500 (75 cycles, PE). Re-pooled libraries for step two were sequenced on the Illumina HiSeq2500 in Rapid Run mode to obtain 50 bp and 140 bp PE reads.

### Calculating optimal re-pooling proportions

To calculate optimal re-pooling proportions after shallow sequencing, we first calculated the digital concentration of reads/*μ*L (*R*). For each sample *i* sequenced in step one, *R*_*i*_ is defined as the number of raw sequencing reads per *μ*L of pooled library. The re-pooling proportion for each sample *i* is then calculated using the following formula:
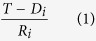
where *T* represents the total number of reads desired for each sample *i* (here 75 M) and *D*_*i*_ represents the number of reads collected for sample *i* in previous runs. Changing the value for *D*_*i*_ and *R*_*i*_ also allows for iterative adjustments of pooling proportions in order to reach the desired total number of reads through multiple re-pooling and sequencing runs.

### RNA-seq data processing and differential gene expression analysis

Sequencing reads were aligned to the reference human genome hg19 using bwa mem^21^ (http://bio-bwa.sourceforge.net). Reads with quality <10 and duplicate reads were removed using samtools rmdup (http://github.com/samtools/). We also removed two samples (barcodes) because the sequencing failed (extremely low number of reads, <1 M). Read counts covering each transcript were calculated using samtools and the Ensembl gene annotations for 57605 genes. Counts data for transcripts with >20 reads were used to run DESeq2^13^. To best account for overdispersion, the DESeq2 model was fit on all sequencing data simultaneously, rather than pairwise matching of treatments and controls. Each control-treatment pair was then matched from an experimental design matrix, and differentially expressed (DE) genes were determined as those with at least one transcript with a Benjamini-Hochberg controlled FDR^14^ (BH-FDR) of 10%. For step two, reads from multiple runs were merged after alignment (at the bam stage) and prior to applying any filter. Reads obtained in step one were not pooled with reads obtained in step two.

To perform hierarchical clustering of the expression levels across treatments, for each transcript in the Ensembl annotations, we calculated FPKMs from the number of reads covering the transcript. To control for potential confounders of expression data, a linear model was used to regress out effects from GC content, transcript length, and an interaction term between GC content and transcript length. These residuals were quantile normalized within each sample, and normalized within each individual by subtracting that individual’s average value per transcript across all treatments. This was calculated after removing the top and bottom deciles of data, usually referred to as 10% trimmed mean or Tukey’s mean. The procedure is implemented in R “mean” function using the “trim = 0.1” option.

The downsampling of reads from shallow sequencing to test the limits of highly multiplexing approaches was performed using the samtools command “view” with the “sub-sampling” option.

### Viability Assays

To assess cell viability in response to the treatment panel, cells were exposed to each environmental stimulus and subsequently evaluated using the CellTiter-Glo Luminescent Assay (Promega Cat-G7570). LCLs were cultured and treated as described above, with the exception of being seeded into a 96-Well-Black tissue culture plate (Fisher). Treated plates were then incubated for 48 hours. After each incubation period, the CellTiter-Glo assay was performed according to the manufacturer protocol. The plate was then scanned in the Fluoroskan Ascent FL plate reader and luminescent signal acquired. For each treatment and control sample, at each time point, experiments were performed in triplicates on one LCL sample. Significant differential viability was assessed by a *t*-test comparing each treatment to the appropriate vehicle-control.

## Additional Information

**How to cite this article**: Moyerbrailean, G. A. *et al.* A high-throughput RNA-seq approach to profile transcriptional responses. *Sci. Rep.* **5**, 14976; doi: 10.1038/srep14976 (2015).

## Supplementary Material

Supplementary Information

## Figures and Tables

**Table 1:** Treatments used in step one.

**Treatment**	**Common Name**	**Control**	**Concentration**†
Ascorbic acid	Vitamin C	Media	1.00 × 10^−5^
Biotin	Biotin	Media	4.75 × 10^−10^
Nicotinic Acid	Vitamin B3	Media	1.50 × 10^−5^
Pantothenic Acid	Vitamin B5	Media	1.00 × 10^−7^
Pyridoxine	Vitamin B6	Media	1.00 × 10^−5^
Retinoic Acid	Vitamin A	Ethanol	1.00 × 10^−8^
Tocopherol	Vitamin E	Ethanol	5.00 × 10^−5^
Plumbagin	Vitamin K3	Ethanol	1.00 × 10^−6^
Aldosterone	Aldosterone	Ethanol	1.00 × 10^−5^
Progesterone (C1)	Progesterone (C1)	DMSO	1.00 × 10^−6^
Progesterone (C2)	Progesterone (C2)	Ethanol	1.00 × 10^−5^
Beta-Estradiol	Estrogen	Ethanol	1.00 × 10^−5^
Dexamethasone	Dexamethasone	Ethanol	1.00 × 10^−5^
Caffeine	Caffeine	Media	1.16 × 10^−3^
Nicotine	Nicotine	Media	6.16 × 10^−4^
Copper (II) Chloride	Copper	Media	6.00 × 10^−5^
Iron (III) Chloride	Iron	Media	5.00 × 10^−3^
Manganese (II) Chloride	Manganese	Media	3.00 × 10^−3^
Molybdenum (V) Chloride	Molybdenum	Media	5.00 × 10^−4^
Sodium Selenite	Selenium	Media	1.00 × 10^−5^
Zinc Chloride	Zinc	Media	8.00 × 10^−5^
Tunicamycin	Tunicamycin	DMSO	2 *μ*g/mL
PM 2.5 (Detroit)	PM 2.5	Media	5 *μ*g/mL

The control for each treatment is the vehicle that was used to dilute it. For example, dexamethasone was prepared from a powder diluted in EtOH, so we used EtOH as control for the dexamethasone treatment. Note that we also matched the concentration of the vehicle used. In the case of all the treatments with EtOH as control, both the treatment and the control wells received 1 ul of EtOH per 10,000 ul of culturing media.

^†^All concentrations are in molarity (M) unless otherwise specified.

**Table 2:** Differentially expressed genes identified in step two.

	**#DEG**† **(% total)**		
		**Shallow**	**Deep**
Copper	Up	758 (6.73%)	1806 (11.43%)
	Down	769 (6.82%)	2167 (13.71%)
Iron	Up	7754 (27.56%)	12465 (41.48%)
	Down	3133 (11.13%)	6134 (20.41%)
Selenium	Up	3198 (21.24%)	5937 (24.01%)
	Down	2535 (16.83%)	5266 (21.29%)
VitaminA	Up	2156 (11.15%)	3337 (14.82%)
	Down	1239 (6.41%)	2553 (11.34%)

^†^10% BH-FDR.
