# Supplementary material for: A high-throughput RNA-seq approach to profile transcriptional responses
Source: Sci Rep. 2015 Oct 29;5:14976. doi: 10.1038/srep14976 (PMC4625130; doi:10.1038/srep14976)
Supplement: Supplementary Information [file srep14976-s1.pdf]

Supplemental Material for: A high-throughput RNA-seq approach to profile transcriptional responses

G. A. Moyerbrailean[1], G. O. Davis[1],
C. T. Harvey[1], D. Watza[1], X. Wen[2], R. Pique-Regi[1,3,*], F. Luca[1,3,*],

[1]Wayne State University, Center for Molecular Medicine and Genetics, Detroit MI, 48201, USA
[2]University of Michigan, Department of Biostatistics, Ann Arbor MI, 48109, USA
[3]Wayne State University, Department of Obstetrics and Gynecology, Detroit MI, 48201, USA

[*]To whom correspondence should be addressed: fluca@wayne.edu, rpique@wayne.edu

Table S1: GO categories for genes upregulated by retinoic acid. Categories in bold are enriched in the deep sequencing data only.

**Biological Process**	**Adj p value**
immune response	1.20×10^{-09}
**translational elongation**	6.00×10^{-05}
cell activation	1.40×10^{-04}
leukocyte activation	4.90×10^{-04}
lymphocyte activation	8.10×10^{-04}
defense response	5.70×10^{-03}
T cell activation	7.70×10^{-03}
positive regulation of biosynthetic process	8.40×10^{-03}
response to oxygen levels	1.20×10^{-02}
positive regulation of cellular biosynthetic process	1.60×10^{-02}
**translation**	1.70×10^{-02}
innate immune response	1.90×10^{-02}
positive regulation of macromolecule biosynthetic process	2.10×10^{-02}
regulation of programmed cell death	2.20×10^{-02}
positive regulation of immune system process	2.20×10^{-02}
response to nutrient	2.20×10^{-02}
regulation of apoptosis	2.30×10^{-02}
positive regulation of cytokine biosynthetic process	2.30×10^{-02}
inflammatory response	2.30×10^{-02}
regulation of cell death	2.40×10^{-02}
response to hypoxia	2.50×10^{-02}
negative regulation of transcription from RNA polymerase II promoter	2.60×10^{-02}
regulation of cytokine production	2.60×10^{-02}
**positive regulation of macromolecule metabolic process**	3.00×10^{-02}
positive regulation of RNA metabolic process	3.30×10^{-02}
**response to organic cyclic substance**	3.50×10^{-02}
response to wounding	3.60×10^{-02}
positive regulation of transcription, DNA-dependent	3.70×10^{-02}
response to vitamin	3.90×10^{-02}
positive regulation of transcription	4.40×10^{-02}
negative regulation of programmed cell death	4.60×10^{-02}
response to extracellular stimulus	4.60×10^{-02}
regulation of transcription from RNA polymerase II promoter	4.70×10^{-02}
positive regulation of gene expression	4.80×10^{-02}
negative regulation of apoptosis	4.80×10^{-02}
negative regulation of cell death	4.90×10^{-02}

Table S2: GO categories for genes downregulated by retinoic acid. Categories in bold are enriched in the deep sequencing data only.

**Biological Process**	**Adj p value**
cell cycle phase	2.20×10^{-03}
cell cycle	7.80×10^{-03}
**hemopoiesis**	8.00×10^{-03}
**response to DNA damage stimulus**	8.20×10^{-03}
**DNA metabolic process**	8.90×10^{-03}
**M phase**	8.90×10^{-03}
**intracellular signaling cascade**	9.20×10^{-03}
**cell cycle process**	9.70×10^{-03}
**immune system development**	1.00×10^{-02}
**hemopoietic or lymphoid organ development**	1.00×10^{-02}
**DNA repair**	1.30×10^{-02}
**cellular response to stress**	1.40×10^{-02}
cell surface receptor linked signal transduction	1.70×10^{-02}

Table S3: GO categories for genes upregulated by copper. Categories in bold are enriched in the deep sequencing data only.

Biological Process	Adj p value
negative regulation of protein ubiquitination	2.00×10^{-09}
ubiquitin-dependent protein catabolic process	3.60×10^{-09}
positive regulation of ubiquitin-protein ligase activity during mitotic cell cycle	4.50×10^{-09}
negative regulation of ubiquitin-protein ligase activity during mitotic cell cycle	4.80×10^{-09}
anaphase-promoting complex-dependent proteasomal ubiquitin-dependent	
protein catabolic process	4.80×10^{-09}
positive regulation of ligase activity	4.90×10^{-09}
positive regulation of ubiquitin-protein ligase activity	6.00×10^{-09}
regulation of ubiquitin-protein ligase activity during mitotic cell cycle	7.20×10^{-09}
negative regulation of ligase activity	7.50×10^{-09}
negative regulation of ubiquitin-protein ligase activity	7.50×10^{-09}
cellular macromolecule catabolic process	1.50×10^{-08}
macromolecule catabolic process	1.90×10^{-08}
proteolysis involved in cellular protein catabolic process	2.30×10^{-08}
cellular protein catabolic process	2.40×10^{-08}
protein catabolic process	2.50×10^{-08}
modification-dependent macromolecule catabolic process	3.00×10^{-08}
modification-dependent protein catabolic process	3.00×10^{-08}
regulation of ligase activity	5.00×10^{-08}
proteolysis	6.20×10^{-08}
regulation of ubiquitin-protein ligase activity	6.50×10^{-08}
positive regulation of protein ubiquitination	6.50×10^{-08}
proteasomal ubiquitin-dependent protein catabolic process	7.90×10^{-08}
proteasomal protein catabolic process	7.90×10^{-08}
regulation of protein ubiquitination	8.60×10^{-08}
negative regulation of protein modification process	1.40×10^{-06}
**positive regulation of cellular protein metabolic process**	2.20×10^{-06}
**positive regulation of protein metabolic process**	1.30×10^{-05}
negative regulation of cellular protein metabolic process	1.60×10^{-05}
**positive regulation of protein modification process**	1.60×10^{-05}
negative regulation of protein metabolic process	4.10×10^{-05}

Continued on next page

TableS3 – continued from previous page

Biological Process	Adj p value
**positive regulation of catalytic activity**	2.80×10^{-04}
**positive regulation of molecular function**	1.00×10^{-03}
**negative regulation of catalytic activity**	1.50×10^{-03}
negative regulation of molecular function	2.20×10^{-03}
**mitotic cell cycle**	3.80×10^{-03}
**regulation of protein modification process**	5.70×10^{-03}
**regulation of cellular protein metabolic process**	6.30×10^{-03}
**translational elongation**	6.80×10^{-03}
response to unfolded protein	1.10×10^{-02}
**protein folding**	1.80×10^{-02}
**cell cycle process**	3.10×10^{-02}

Table S4: GO categories for genes downregulated by copper. Categories in bold are enriched in the deep sequencing data only.

**Biological Process**	**Adj p value**
M phase of mitotic cell cycle	7.60×10^{-04}
organelle fission	9.20×10^{-04}
mitosis	1.20×10^{-03}
nuclear division	1.20×10^{-03}
M phase	3.30×10^{-03}
**cellular amino acid biosynthetic process**	6.60×10^{-03}
cell cycle phase	1.30×10^{-02}
**amine biosynthetic process**	1.50×10^{-02}
**cell proliferation**	4.30×10^{-02}
**nitrogen compound biosynthetic process**	4.90×10^{-02}
**immune response**	4.90×10^{-02}

Table S5: GO categories for genes unregulated by iron. Categories in bold are enriched in the deep sequencing data only.

Biological Process	Adj p value
biological adhesion	8.70×10^{-27}
cell adhesion	1.00×10^{-26}
ion transport	1.10×10^{-17}
neurological system process	1.10×10^{-14}
metal ion transport	1.40×10^{-12}
cell-cell adhesion	5.90×10^{-12}
cell-cell signaling	9.50×10^{-12}
cell surface receptor linked signal transduction	9.80×10^{-11}
synaptic transmission	4.70×10^{-10}
cell projection organization	1.10×10^{-09}
cation transport	3.00×10^{-09}
cell morphogenesis	3.50×10^{-09}
cell morphogenesis involved in differentiation	7.90×10^{-09}
transmission of nerve impulse	9.20×10^{-09}
axonogenesis	1.50×10^{-08}
cellular component morphogenesis	3.30×10^{-08}
neuron differentiation	4.70×10^{-08}
neuron projection morphogenesis	4.80×10^{-08}
neuron projection development	4.80×10^{-08}
cell morphogenesis involved in neuron differentiation	5.80×10^{-08}
neuron development	6.20×10^{-08}
extracellular structure organization	1.30×10^{-07}
cell projection morphogenesis	1.40×10^{-07}
enzyme linked receptor protein signaling pathway	1.60×10^{-07}
**regulation of system process**	3.00×10^{-07}
cognition	6.10×10^{-07}
cell part morphogenesis	6.40×10^{-07}
homophilic cell adhesion	4.40×10^{-06}
cell motion	5.10×10^{-06}
transmembrane receptor protein tyrosine kinase signaling pathway	6.80×10^{-06}
**sodium ion transport**	6.90×10^{-06}
regulation of small GTPase mediated signal transduction	1.30×10^{-05}
monovalent inorganic cation transport	1.40×10^{-05}
behavior	3.60×10^{-05}
potassium ion transport	3.80×10^{-05}

Continued on next page

TableS5 – continued from previous page

**Biological Process**	**Adj p value**
**learning or memory**	5.20×10^{-05}
localization of cell	5.70×10^{-05}
cell motility	5.70×10^{-05}
protein amino acid phosphorylation	5.80×10^{-05}
anion transport	7.60×10^{-05}
calcium ion transport	8.20×10^{-05}
extracellular matrix organization	1.30×10^{-04}
**excretion**	1.90×10^{-04}
**chemical homeostasis**	1.90×10^{-04}
**ion homeostasis**	3.90×10^{-04}
**muscle contraction**	4.20×10^{-04}
**transmembrane transport**	4.50×10^{-04}
di-, tri-valent inorganic cation transport	5.20×10^{-04}
axon guidance	5.60×10^{-04}
regulation of Ras protein signal transduction	6.40×10^{-04}
**muscle system process**	6.40×10^{-04}
**G-protein coupled receptor protein signaling pathway**	7.80×10^{-04}
**cell migration**	1.30×10^{-03}
**organic acid transport**	2.60×10^{-03}
**carboxylic acid transport**	2.60×10^{-03}
**sensory perception**	2.80×10^{-03}
**wound healing**	2.80×10^{-03}
**integrin-mediated signaling pathway**	2.90×10^{-03}
**memory**	3.00×10^{-03}
inorganic anion transport	3.10×10^{-03}
regulation of Rho protein signal transduction	3.70×10^{-03}
**heart development**	3.90×10^{-03}
phosphate metabolic process	3.90×10^{-03}
phosphorus metabolic process	3.90×10^{-03}
**regulation of synaptic transmission**	5.40×10^{-03}
**regulation of transmission of nerve impulse**	6.30×10^{-03}
**regulation of cell morphogenesis**	6.30×10^{-03}
**cellular chemical homeostasis**	7.40×10^{-03}
**multicellular organismal response to stress**	8.60×10^{-03}
**cellular ion homeostasis**	9.00×10^{-03}
**sensory perception of mechanical stimulus**	9.40×10^{-03}
**regulation of neurological system process**	9.50×10^{-03}
**cAMP-mediated signaling**	1.10×10^{-02}

Continued on next page

Biological Process	Adj p value
**cyclic-nucleotide-mediated signaling**	1.10×10^{-02}
**positive regulation of lyase activity**	1.10×10^{-02}
limb morphogenesis	1.30×10^{-02}
appendage morphogenesis	1.30×10^{-02}
**embryonic morphogenesis**	1.40×10^{-02}
**cation homeostasis**	1.40×10^{-02}
**regulation of lyase activity**	1.60×10^{-02}
actin filament-based process	1.70×10^{-02}
**positive regulation of cyclase activity**	1.70×10^{-02}
embryonic appendage morphogenesis	1.70×10^{-02}
embryonic limb morphogenesis	1.70×10^{-02}
**secretion**	1.80×10^{-02}
**adult behavior**	1.90×10^{-02}
**vasculature development**	1.90×10^{-02}
**G-protein signaling, coupled to cyclic nucleotide second messenger**	1.90×10^{-02}
sensory organ development	2.00×10^{-02}
limb development	2.00×10^{-02}
appendage development	2.00×10^{-02}
**response to wounding**	2.00×10^{-02}
**regulation of cyclase activity**	2.10×10^{-02}
**G-protein signaling, coupled to cAMP nucleotide second messenger**	2.10×10^{-02}
**gland development**	2.20×10^{-02}
**sensory perception of light stimulus**	2.30×10^{-02}
**visual perception**	2.30×10^{-02}
**positive regulation of adenylate cyclase activity**	2.30×10^{-02}
**muscle cell differentiation**	2.30×10^{-02}
**ear development**	2.60×10^{-02}
**inner ear development**	2.70×10^{-02}
actin cytoskeleton organization	2.70×10^{-02}
**sensory perception of sound**	2.70×10^{-02}
blood vessel development	2.70×10^{-02}
synapse organization	2.90×10^{-02}
**startle response**	3.10×10^{-02}
**cell-substrate adhesion**	3.10×10^{-02}
**cell junction organization**	3.20×10^{-02}
**activation of adenylate cyclase activity**	3.30×10^{-02}

Continued on next page

TableS5 – continued from previous page

Biological Process	Adj p value
**regulation of locomotion**	3.40×10^{-02}
**cell-matrix adhesion**	3.40×10^{-02}
**striated muscle tissue development**	3.50×10^{-02}
**tube development**	3.50×10^{-02}
**urogenital system development**	3.50×10^{-02}
chloride transport	3.50×10^{-02}
adult locomotory behavior	3.50×10^{-02}
**regulation of cell adhesion**	3.80×10^{-02}
**regulation of heart contraction**	4.00×10^{-02}
**second-messenger-mediated signaling**	4.40×10^{-02}
**neuromuscular process**	4.40×10^{-02}
**regulation of cyclic nucleotide biosynthetic process**	4.40×10^{-02}
**regulation of nucleotide biosynthetic process**	4.40×10^{-02}
**regulation of cell migration**	4.60×10^{-02}
**regulation of cyclic nucleotide metabolic process**	4.60×10^{-02}
**regulation of adenylate cyclase activity**	4.80×10^{-02}
**ectoderm development**	4.80×10^{-02}
**sperm motility**	4.80×10^{-02}

Table S6: GO categories for genes downregulated by iron. Categories in bold are enriched in the deep sequencing data only.

Biological Process	Adj p value
RNA processing	$7.70{\times}10^{-13}$
RNA splicing	$1.00{\times}10^{-12}$
organelle fission	$1.70{\times}10^{-11}$
nuclear division	$7.50{\times}10^{-11}$
mitosis	$7.50{\times}10^{-11}$
M phase of mitotic cell cycle	$9.90{\times}10^{-11}$
mRNA processing	$1.10{\times}10^{-10}$
mRNA metabolic process	$1.30{\times}10^{-10}$
DNA metabolic process	$1.40{\times}10^{-10}$
cell cycle	$3.60{\times}10^{-10}$
M phase	$1.30{\times}10^{-09}$
cell cycle process	$9.60{\times}10^{-09}$
mitotic cell cycle	$9.90{\times}10^{-09}$
cell cycle phase	$6.70{\times}10^{-08}$
RNA splicing, via transesterification reactions with bulged adenosine as nucleophile	$1.10{\times}10^{-07}$
RNA splicing, via transesterification reactions	$1.10{\times}10^{-07}$
nuclear mRNA splicing, via spliceosome	$1.10{\times}10^{-07}$
cell division	$1.10{\times}10^{-07}$
response to DNA damage stimulus	$4.50{\times}10^{-07}$
cellular response to stress	$2.10{\times}10^{-06}$
DNA repair	$8.00{\times}10^{-06}$
**chromosome organization**	$8.20{\times}10^{-06}$
chromosome segregation	$2.90{\times}10^{-05}$
cellular macromolecule catabolic process	$6.60{\times}10^{-05}$
**protein modification by small protein conjugation or removal**	$7.10{\times}10^{-05}$
response to virus	$1.50{\times}10^{-04}$
protein targeting to membrane	$2.30{\times}10^{-04}$
macromolecule catabolic process	$3.30{\times}10^{-04}$
**regulation of cell cycle**	$3.40{\times}10^{-04}$
protein transport	$6.00{\times}10^{-04}$
establishment of protein localization	$8.70{\times}10^{-04}$
proteolysis involved in cellular protein catabolic process	$1.00{\times}10^{-03}$
protein catabolic process	$1.30{\times}10^{-03}$

Continued on next page

TableS6 – continued from previous page

**Biological Process**	**Adj p value**
modification-dependent macromolecule catabolic process	1.30×10^{-03}
modification-dependent protein catabolic process	1.30×10^{-03}
protein localization	1.40×10^{-03}
cellular protein catabolic process	1.40×10^{-03}
generation of precursor metabolites and energy	3.10×10^{-03}
**apoptosis**	3.70×10^{-03}
**programmed cell death**	4.10×10^{-03}
**cofactor biosynthetic process**	5.30×10^{-03}
intracellular transport	5.70×10^{-03}
DNA replication	7.40×10^{-03}
**protein modification by small protein conjugation**	7.70×10^{-03}
cellular respiration	8.80×10^{-03}
energy derivation by oxidation of organic compounds	9.20×10^{-03}
**chromatin modification**	1.30×10^{-02}
**chromosome condensation**	1.30×10^{-02}
**histone modification**	1.50×10^{-02}
ubiquitin-dependent protein catabolic process	1.60×10^{-02}
**covalent chromatin modification**	1.80×10^{-02}
**RNA elongation**	2.00×10^{-02}
proteasomal ubiquitin-dependent protein catabolic process	2.20×10^{-02}
proteasomal protein catabolic process	2.20×10^{-02}
**cell death**	2.20×10^{-02}
**cofactor metabolic process**	2.30×10^{-02}
**DNA recombination**	2.70×10^{-02}
**regulation of mitotic cell cycle**	2.80×10^{-02}
**death**	2.90×10^{-02}
cellular protein localization	3.20×10^{-02}
**mitotic sister chromatid segregation**	3.40×10^{-02}
cotranslational protein targeting to membrane	4.10×10^{-02}
**chromatin organization**	4.10×10^{-02}
**RNA elongation from RNA polymerase II promoter**	4.20×10^{-02}
cellular macromolecule localization	4.30×10^{-02}
**negative regulation of macromolecule metabolic process**	4.80×10^{-02}
**microtubule organizing center organization**	4.90×10^{-02}
**sister chromatid segregation**	4.90×10^{-02}

Table S7: GO categories for genes upregulated by selenium. Categories in bold are enriched in the deep sequencing data only.

**Biological Process**	**Adj p value**
translation	7.40×10^{-43}
translational elongation	4.90×10^{-32}
RNA processing	8.20×10^{-18}
RNA splicing	4.40×10^{-12}
anaphase-promoting complex-dependent proteasomal ubiquitin-dependent protein catabolic process	4.40×10^{-12}
negative regulation of ubiquitin-protein ligase activity during mitotic cell cycle	4.40×10^{-12}
negative regulation of ligase activity	4.70×10^{-12}
negative regulation of ubiquitin-protein ligase activity	4.70×10^{-12}
positive regulation of ubiquitin-protein ligase activity	7.20×10^{-12}
positive regulation of ubiquitin-protein ligase activity during mitotic cell cycle	7.30×10^{-12}
ribonucleoprotein complex biogenesis	9.30×10^{-12}
negative regulation of protein ubiquitination	1.00×10^{-11}
regulation of ubiquitin-protein ligase activity during mitotic cell cycle	1.10×10^{-11}
positive regulation of ligase activity	5.30×10^{-11}
regulation of ubiquitin-protein ligase activity	6.80×10^{-11}
proteasomal protein catabolic process	1.60×10^{-10}
proteasomal ubiquitin-dependent protein catabolic process	1.60×10^{-10}
mRNA processing	1.70×10^{-10}
regulation of ligase activity	4.80×10^{-10}
mRNA metabolic process	6.90×10^{-10}
RNA splicing, via transesterification reactions with bulged adenosine as nucleophile	7.10×10^{-10}
RNA splicing, via transesterification reactions	7.10×10^{-10}
nuclear mRNA splicing, via spliceosome	7.10×10^{-10}
positive regulation of protein ubiquitination	7.90×10^{-10}
ribosome biogenesis	2.90×10^{-09}
generation of precursor metabolites and energy	1.50×10^{-08}
regulation of protein ubiquitination	1.50×10^{-08}
ncRNA metabolic process	2.90×10^{-08}
cellular macromolecular complex subunit organization	3.10×10^{-08}
cellular macromolecular complex assembly	5.30×10^{-08}

Continued on next page

TableS7 – continued from previous page

Biological Process	Adj p value
rRNA metabolic process	2.00×10^{-07}
rRNA processing	3.50×10^{-07}
**electron transport chain**	1.10×10^{-06}
**oxidative phosphorylation**	1.90×10^{-06}
ubiquitin-dependent protein catabolic process	3.80×10^{-06}
**ncRNA processing**	8.30×10^{-06}
macromolecular complex assembly	1.30×10^{-05}
**negative regulation of cellular protein metabolic process**	1.40×10^{-05}
**macromolecular complex subunit organization**	1.80×10^{-05}
**negative regulation of protein metabolic process**	3.10×10^{-05}
nucleosome assembly	3.20×10^{-05}
chromatin assembly	3.60×10^{-05}
**translational initiation**	3.70×10^{-05}
nucleosome organization	7.00×10^{-05}
protein-DNA complex assembly	7.00×10^{-05}
**negative regulation of protein modification process**	8.10×10^{-05}
DNA metabolic process	1.50×10^{-04}
**cellular respiration**	3.30×10^{-04}
**DNA replication**	6.90×10^{-04}
**ribosomal large subunit biogenesis**	2.50×10^{-03}
**mitochondrial electron transport, NADH to ubiquinone**	2.50×10^{-03}
**respiratory electron transport chain**	2.80×10^{-03}
response to DNA damage stimulus	2.90×10^{-03}
**mitochondrial ATP synthesis coupled electron transport**	3.10×10^{-03}
**ATP synthesis coupled electron transport**	3.10×10^{-03}
**nucleic acid transport**	3.60×10^{-03}
**establishment of RNA localization**	3.60×10^{-03}
**RNA transport**	3.60×10^{-03}
ribonucleoprotein complex assembly	4.00×10^{-03}
chromatin assembly or disassembly	4.60×10^{-03}
RNA elongation from RNA polymerase II promoter	6.70×10^{-03}
**energy derivation by oxidation of organic compounds**	7.30×10^{-03}
RNA elongation	7.30×10^{-03}
**RNA localization**	7.70×10^{-03}
**DNA-dependent DNA replication**	8.10×10^{-03}
**mitotic cell cycle**	9.80×10^{-03}
cellular macromolecule catabolic process	1.00×10^{-02}

Continued on next page

TableS7 – continued from previous page

**Biological Process**	**Adj p value**
**DNA packaging**	1.30×10^{-02}
protein folding	1.50×10^{-02}
**nucleobase, nucleoside, nucleotide and nucleic acid transport**	1.50×10^{-02}
negative regulation of catalytic activity	1.80×10^{-02}
**DNA repair**	1.80×10^{-02}
modification-dependent macromolecule catabolic process	2.20×10^{-02}
modification-dependent protein catabolic process	2.20×10^{-02}
**mitochondrial respiratory chain complex I assembly**	2.40×10^{-02}
**NADH dehydrogenase complex assembly**	2.40×10^{-02}
**mRNA transport**	2.80×10^{-02}
**posttranscriptional regulation of gene expression**	2.80×10^{-02}
**spliceosome assembly**	3.00×10^{-02}
proteolysis involved in cellular protein catabolic process	4.40×10^{-02}
**positive regulation of protein modification process**	4.60×10^{-02}
cellular protein catabolic process	4.70×10^{-02}

Table S8: GO categories for genes downregulated by selenium. Categories in bold are enriched in the deep sequencing data only.

Biological Process	Adj p value
**intracellular signaling cascade**	5.10×10^{-04}
**actin cytoskeleton organization**	5.20×10^{-03}
**leukocyte activation**	6.50×10^{-03}
**chromatin modification**	6.80×10^{-03}
**actin filament-based process**	7.60×10^{-03}
**regulation of small GTPase mediated signal transduction**	9.80×10^{-03}
cell activation	1.00×10^{-02}
**protein transport**	1.20×10^{-02}
**regulation of transcription**	1.20×10^{-02}
**establishment of protein localization**	1.30×10^{-02}
lymphocyte activation	1.30×10^{-02}
**Ras protein signal transduction**	1.70×10^{-02}
**glycoprotein biosynthetic process**	1.80×10^{-02}
**protein kinase cascade**	1.80×10^{-02}
**chromatin remodeling**	1.80×10^{-02}
**chromosome organization**	1.80×10^{-02}
**hemopoiesis**	1.80×10^{-02}
**regulation of gene expression, epigenetic**	1.90×10^{-02}
**protein localization**	2.00×10^{-02}
**glycoprotein metabolic process**	2.10×10^{-02}
**transcription**	2.20×10^{-02}
**hemopoietic or lymphoid organ development**	2.90×10^{-02}
**glycosylation**	3.30×10^{-02}
**biopolymer glycosylation**	3.30×10^{-02}
**protein amino acid glycosylation**	3.30×10^{-02}
**actin filament organization**	4.70×10^{-02}
**chromatin organization**	4.70×10^{-02}

Table S9: **Correlation of downsampling analysis to deep sequencing data.** Shown for each treatment is the original correlation between shallow and deep sequencing (Ratio=1, based on transcript log fold change as in Figure S5), as well as the correlation between results calculated using downsampled shallow sequencing reads corresponding to the indicated downsampling ratio.

**Treatment**	**Downsample Ratio**	**Spearman's** ρ	**95% CI**	**# DEG (10% BH-FDR)**
Vitamin A	1	0.734	0.731 - 0.737	3391
	1/2	0.672	0.669 - 0.676	2373
	1/4	0.584	0.580 - 0.588	1522
	1/6	0.539	0.535 - 0.544	1082
	1/8	0.525	0.520 - 0.530	846
	1/10	0.470	0.465 - 0.474	677
	1/12	0.514	0.509 - 0.520	587
Copper	1	0.746	0.742 - 0.749	1526
	1/2	0.691	0.686 - 0.695	742
	1/4	0.647	0.640 - 0.655	257
	1/6	0.590	0.583 - 0.598	149
	1/8	0.559	0.549 - 0.569	93
	1/10	0.520	0.509 - 0.531	76
	1/12	0.505	0.493 - 0.516	56
Iron	1	0.814	0.813 - 0.816	10677
	1/2	0.734	0.732 - 0.737	6908
	1/4	0.641	0.638 - 0.644	3780
	1/6	0.582	0.578 - 0.585	2436
	1/8	0.536	0.531 - 0.540	1788
	1/10	0.505	0.500 - 0.509	1339
	1/12	0.479	0.474 - 0.483	1014
Selenium	1	0.824	0.822 - 0.826	5521
	1/2	0.798	0.795 - 0.801	3314
	1/4	0.753	0.749 - 0.757	1668
	1/6	0.738	0.733 - 0.743	1122
	1/8	0.738	0.732 - 0.744	792
	1/10	0.705	0.699 - 0.712	592
	1/12	0.717	0.709 - 0.724	492

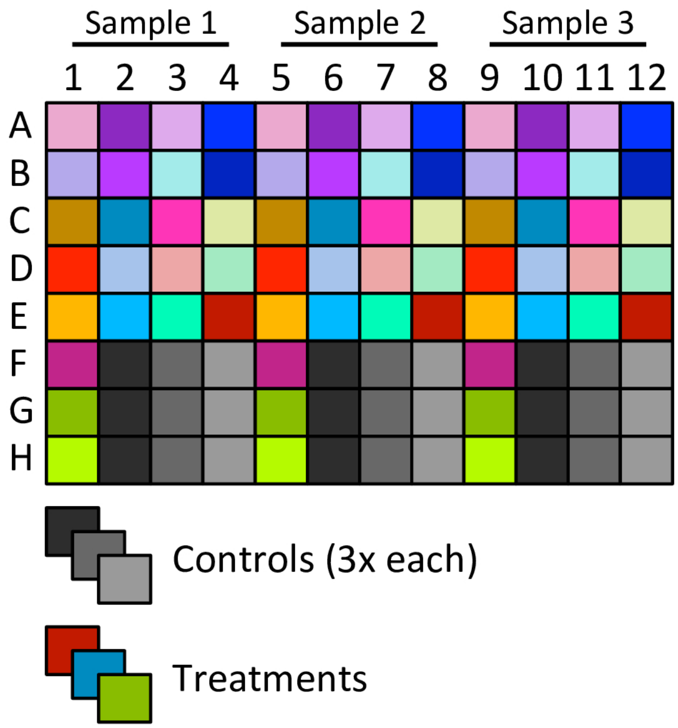

Figure S1: **The 96-well plate design used for this study.** LCLs from three individual samples were treated in parallel on the same plate (sample 1, 2 and 3) to analyze 32 conditions (represented by different colors). The 32 conditions correspond to 23 treatments and 3 vehicle controls. For each LCL sample, each control treatment (in different shades of grey) was performed in triplicates.

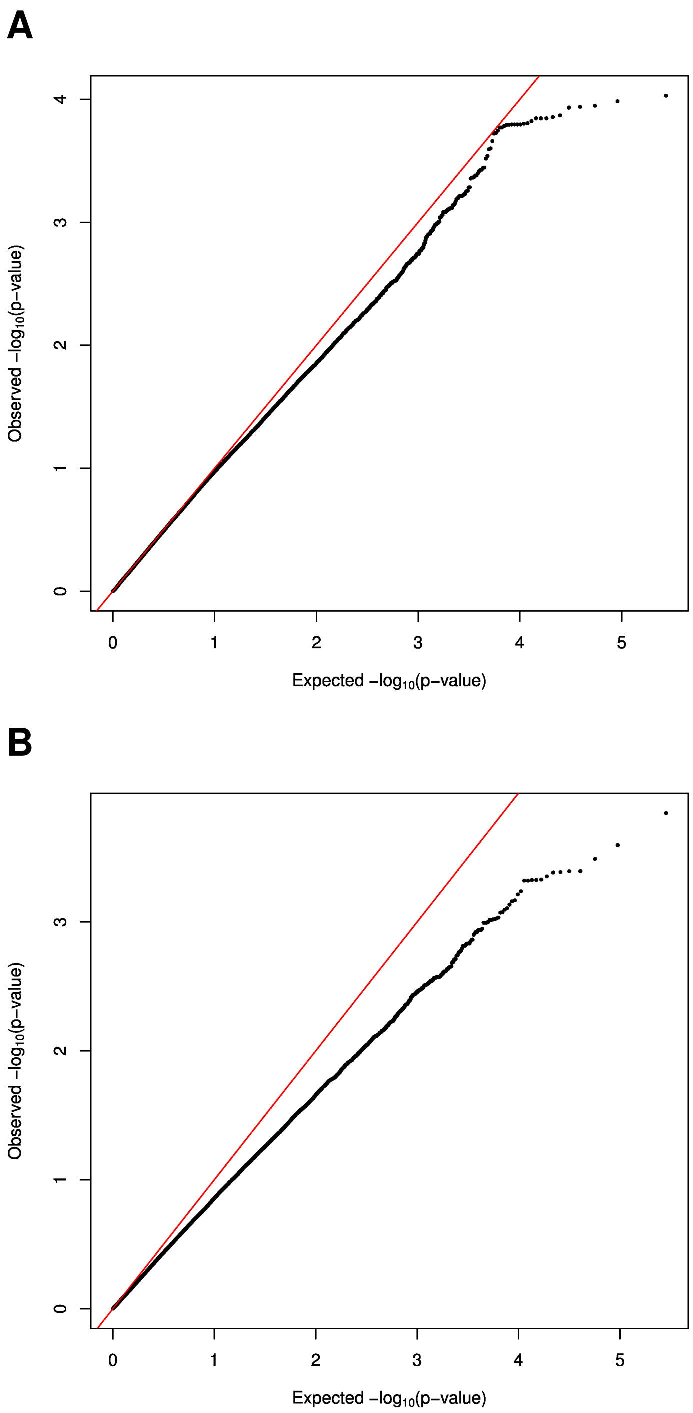

Figure S2: **Comparison of gene expression differences between controls.** Show are QQ-plots of the p-value distribution from DESeq2 comparing media to ethanol from step one (A) and step two (B).

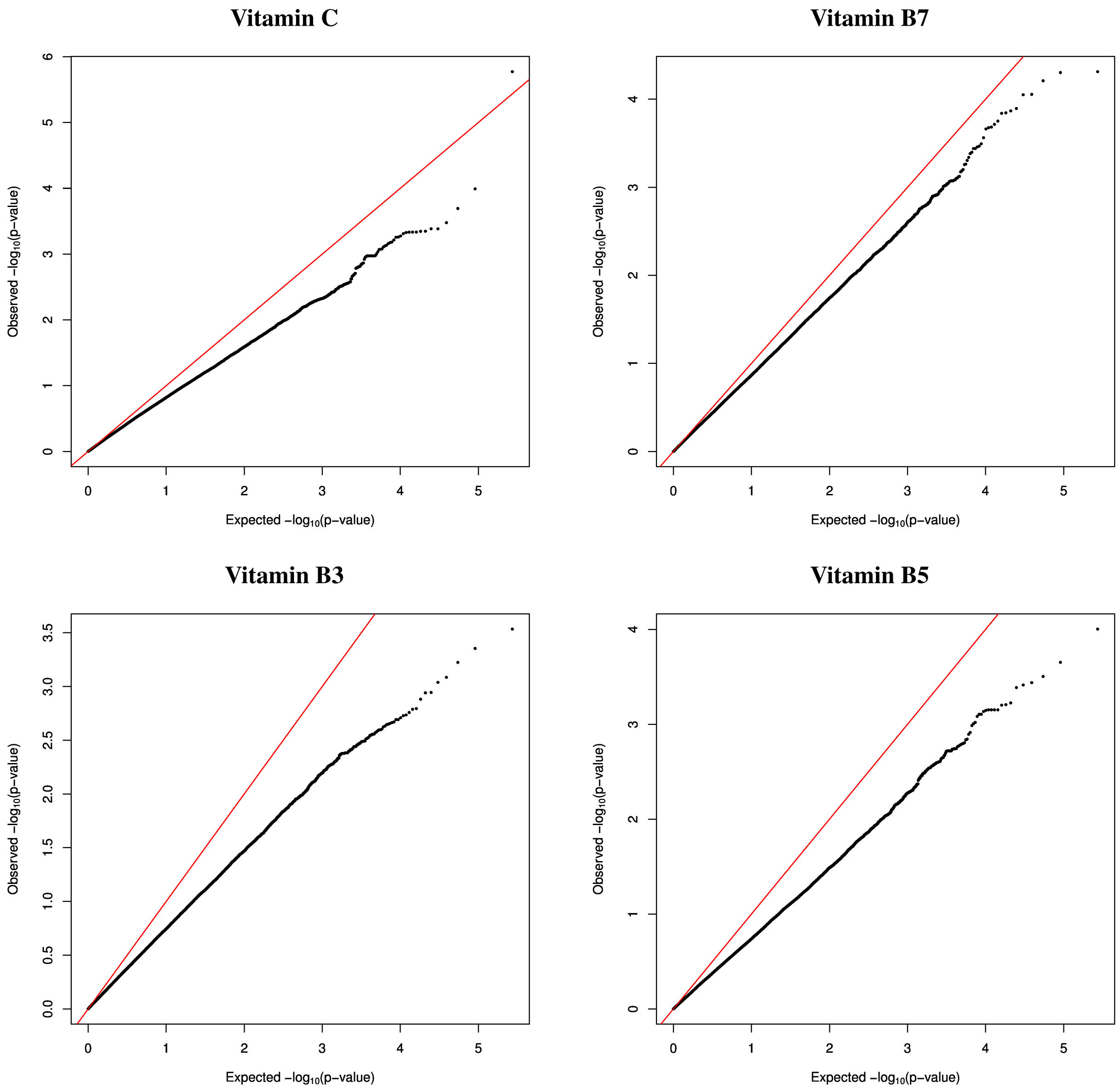

Figure S3: **QQplot of the p-value distribution for DE genes for each step one treatment.** Each treatment was analyzed with respect to the appropriate control (see Table 1).

Figure S3 . . . continued from previous page

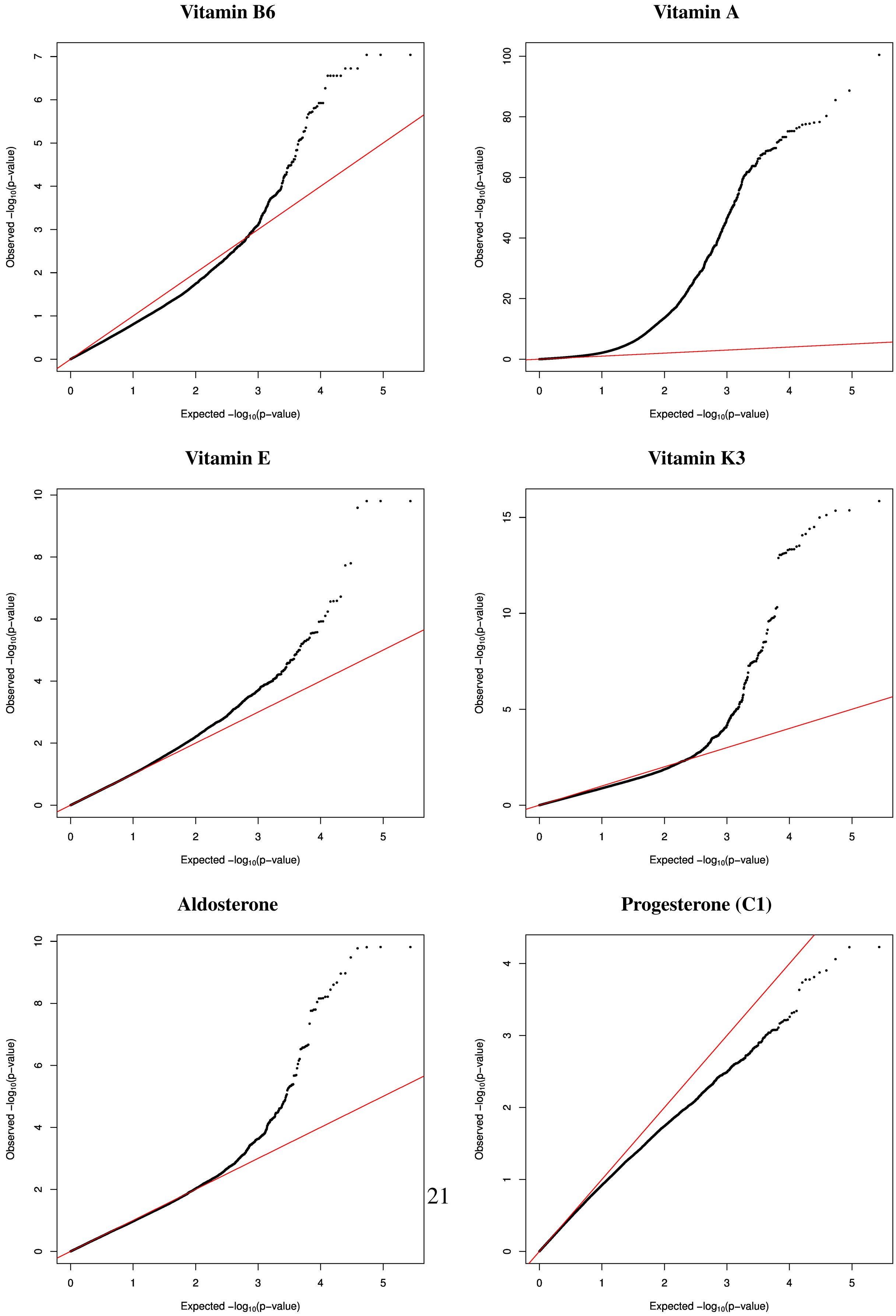

Figure S3 . . . continued from previous page

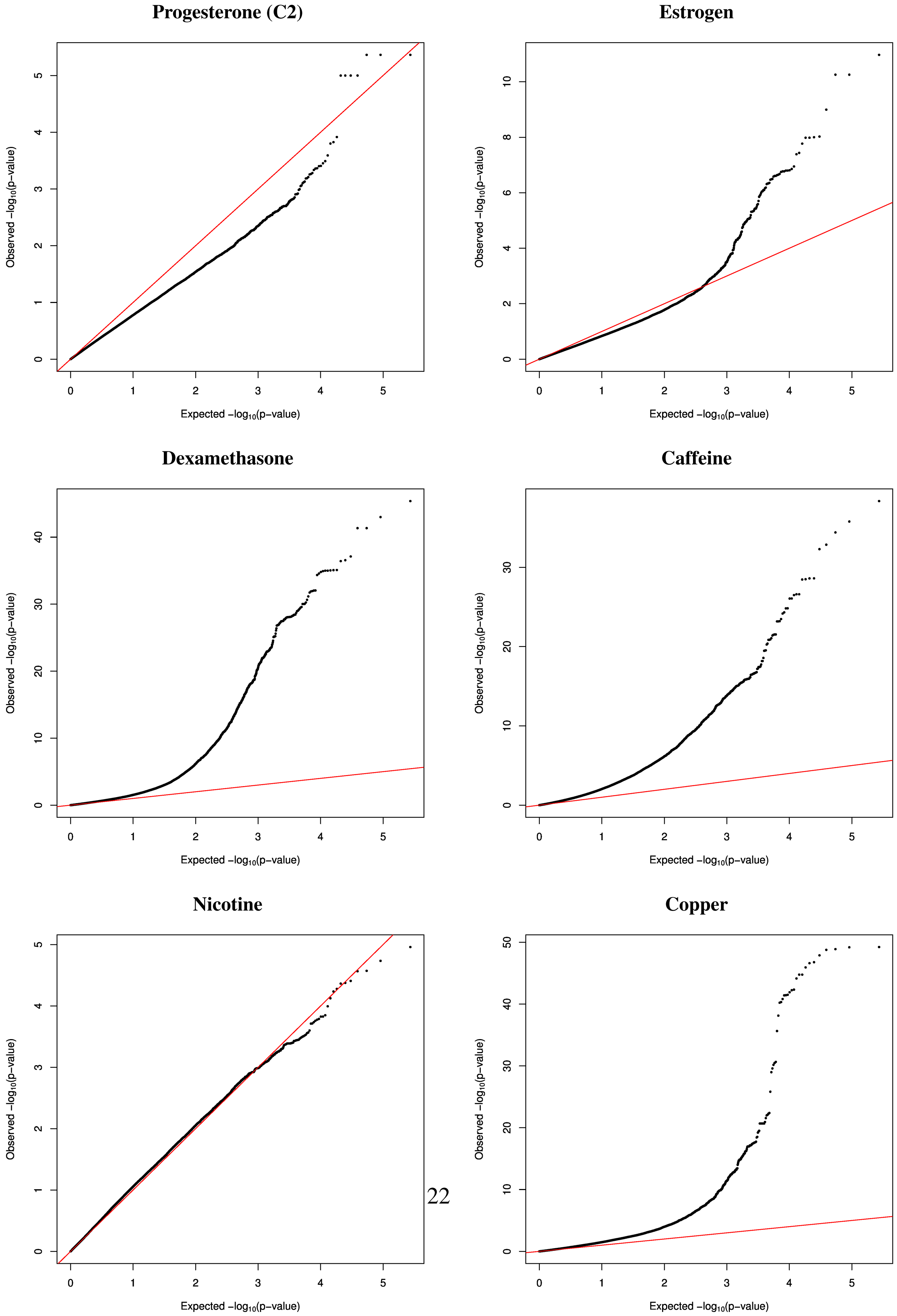

Figure S3 . . . continued from previous page

PM 2.5

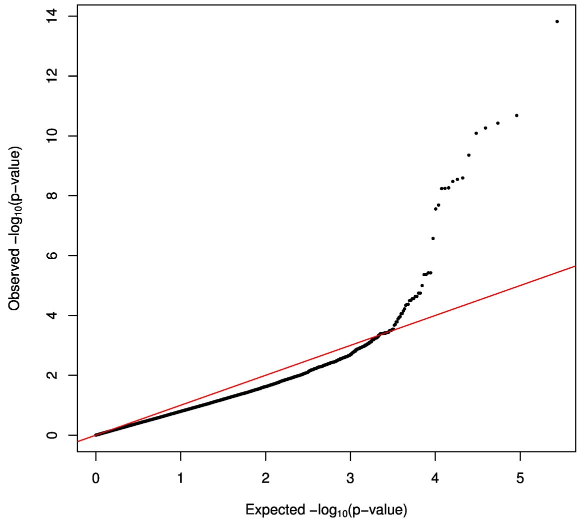

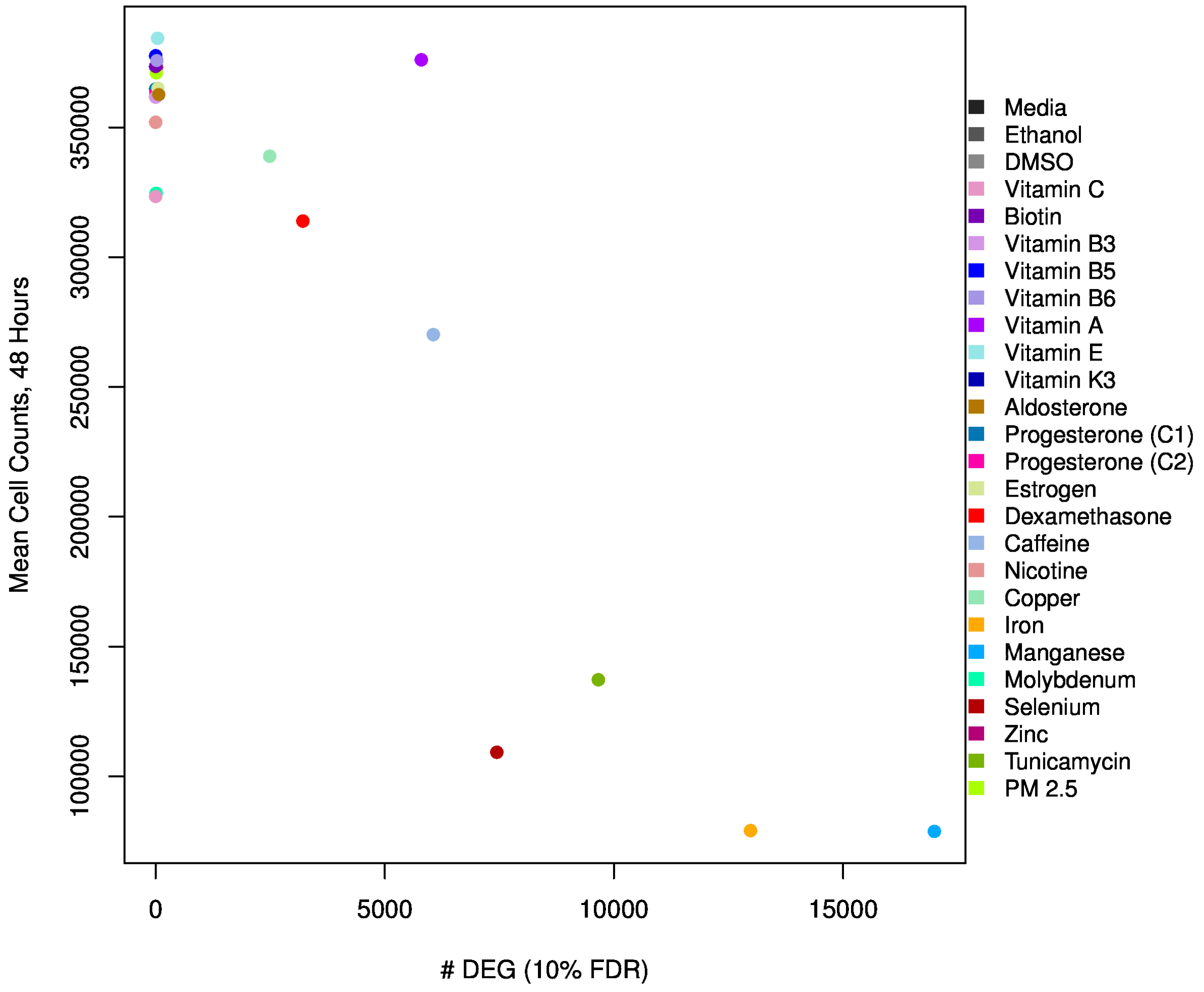

Figure S4: **Differential expression and cell viability.** Each point represents the average viability across three replicates after 48 hours for the indicated treatment. Differentially expressed genes were assessed after a 6-hour treatment, as described in Methods.

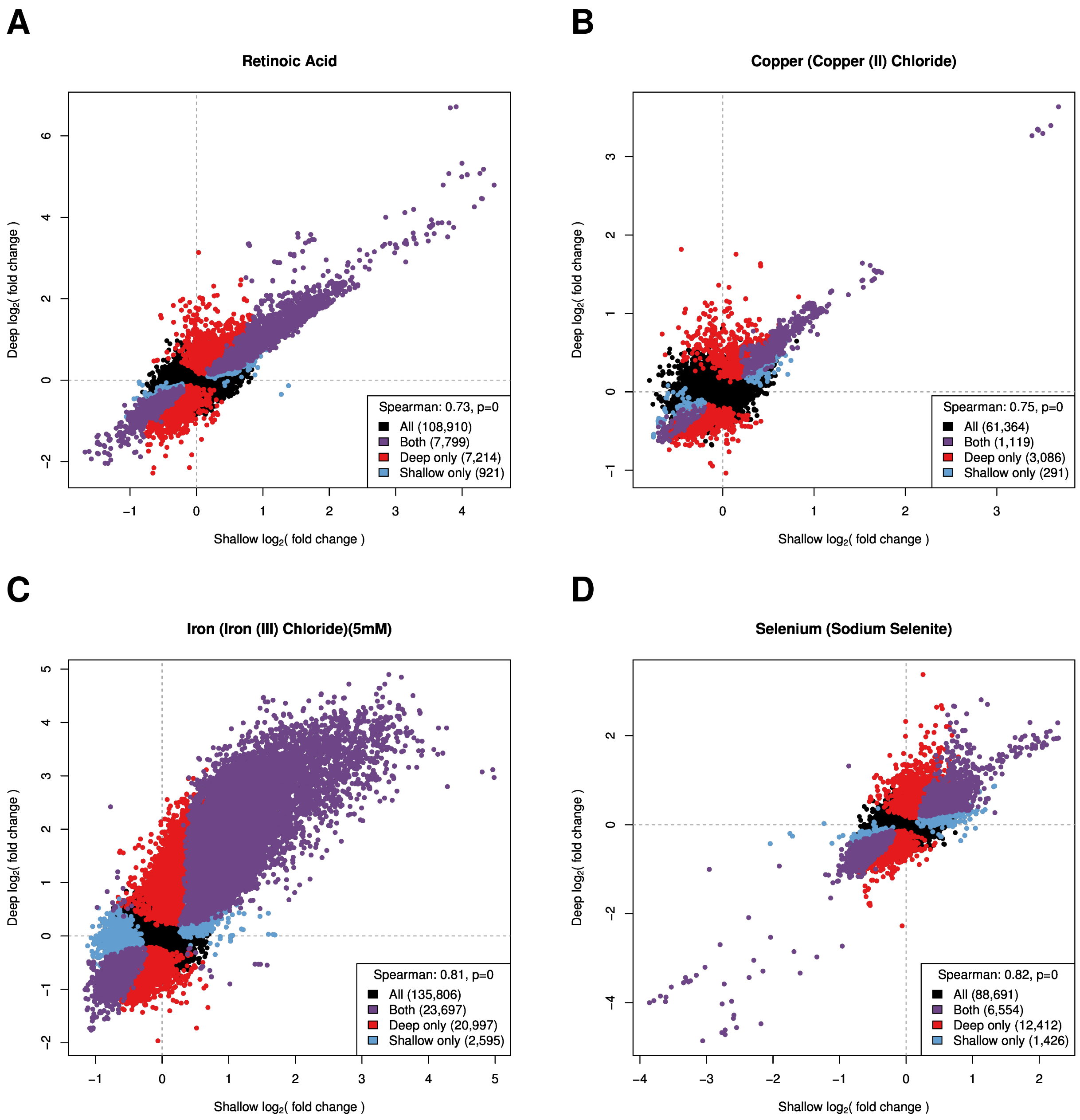

Figure S5: **Correlation in the transcriptional response between shallow and deep sequencing.** Plotted is the $\log_2$(fold change) for each genetranscript calculated from shallow and deep sequencing data for the four treatments analyzed in step two. Colored points represent transcripts differentially expressed at 1% BH-FDR. Vitamin A (A), copper (B), iron (C), selenium (D).

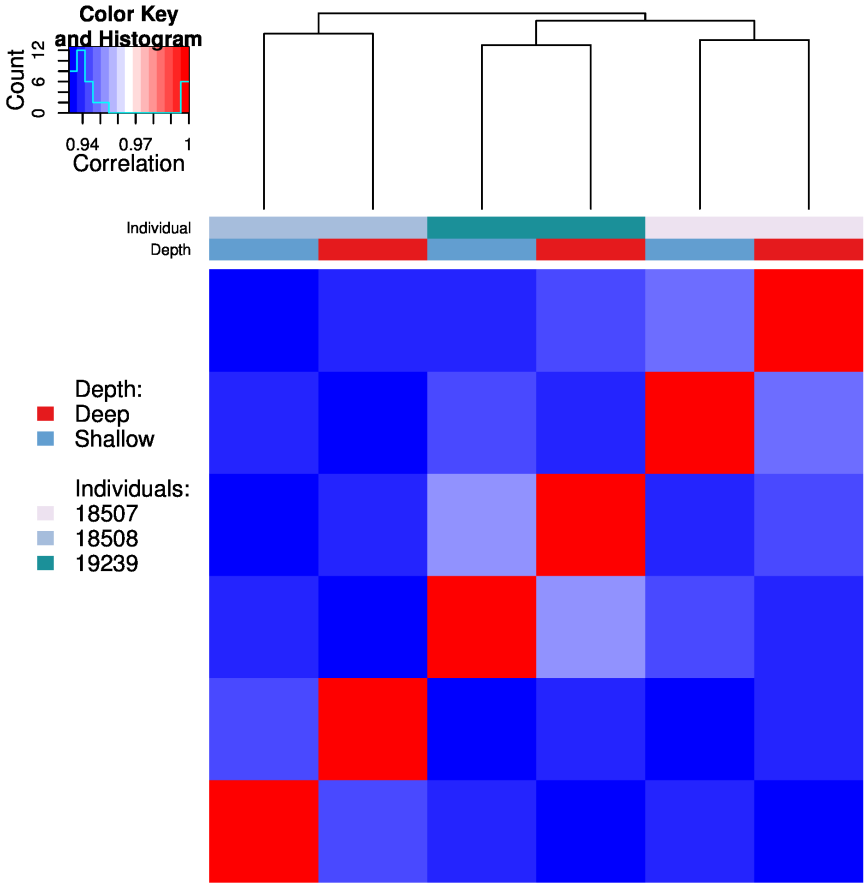

Figure S6: **Comparison of downsampled deep sequencing to shallow sequencing.** Deep sequencing data for the media controls was downsampled to the level of shallow sequencing, and gene expression (FPKM) values were compared. Shallow and downsampled deep samples cluster closely within individual libraries.

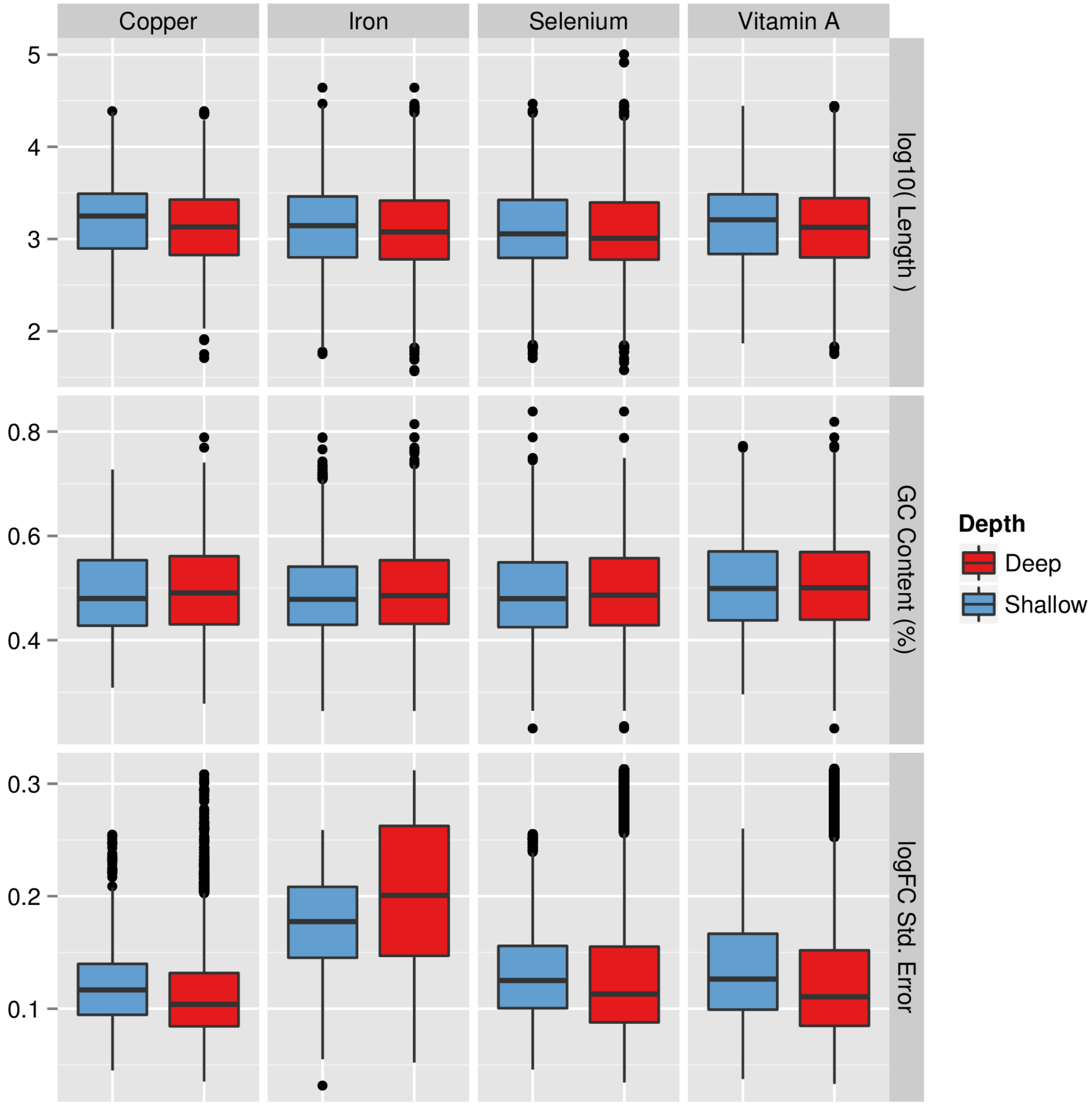

Figure S7: **Features of differentially expressed transcripts.** Shown for each treatment are comparisons between sets of differentially expressed transcripts (10% BH-FDR) for transcript length (in bp), GC content, and the standard error of the log fold change from DESeq2. Transcript length was log_{10} transformed for display purposes.

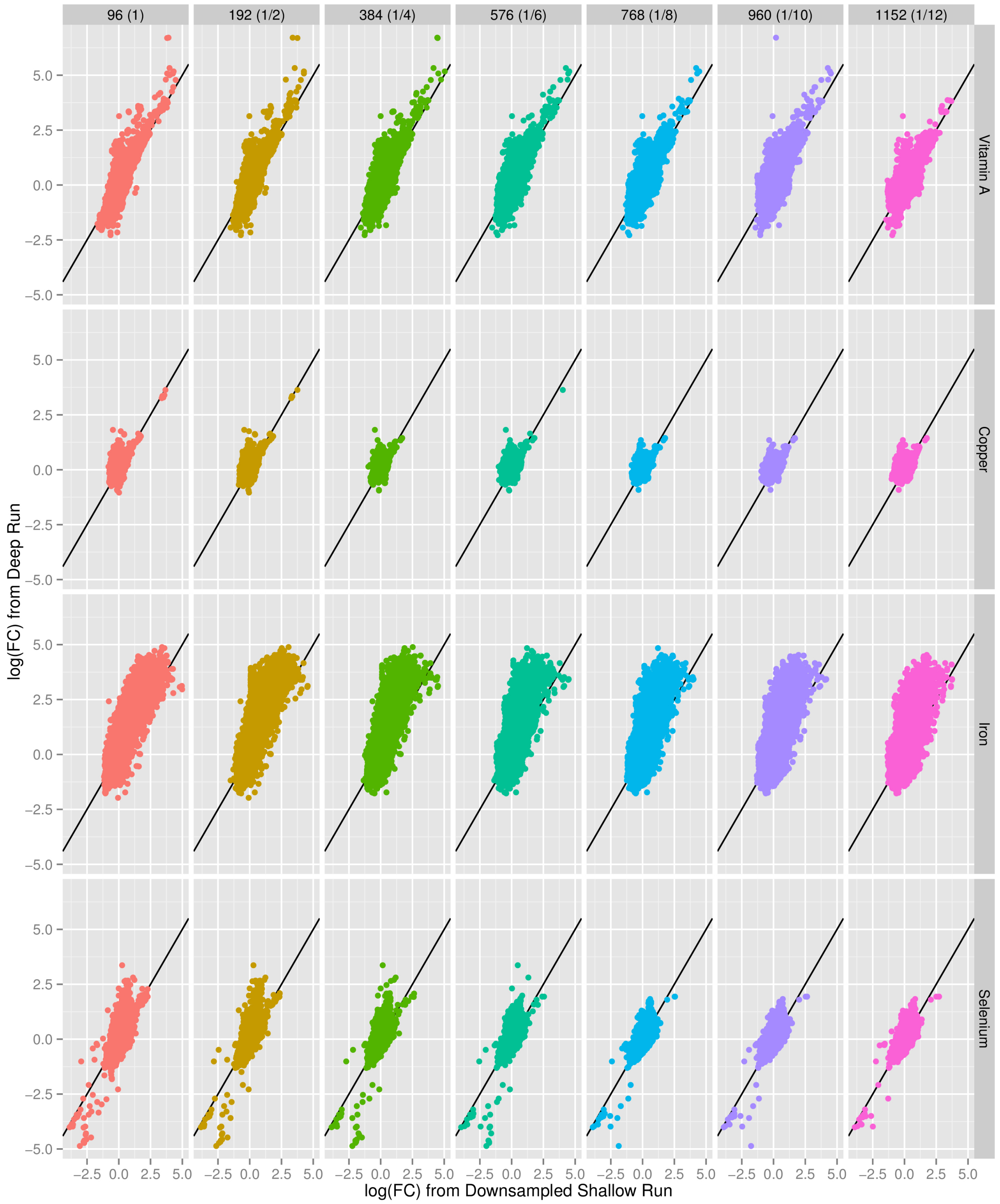

Figure S8: **Simulation of higher multiplexing through downsampling.** Shallow sequencing data for selected treatments were downsampled to reflect indicated multiplex level, then compared to the deep sequencing data. A table of the correlation values can be found in Table S9.
